# A three-criteria performance score for rats exercising on a running treadmill

**DOI:** 10.1371/journal.pone.0219167

**Published:** 2019-07-09

**Authors:** Juan Gabriel Ríos-Kristjánsson, David Rizo-Roca, Karen Mist Kristjánsdóttir, Cristian Andrés Núñez-Espinosa, Joan Ramon Torrella, Teresa Pagès, Ginés Viscor

**Affiliations:** 1 Department of Cell Biology, Physiology & Immunology, Faculty of Biology, University of Barcelona, Barcelona, Spain; 2 Department of Biotechnology and Chemical Engineering, Aarhus University School of Engineering, Aarhus N, Denmark; 3 School of Medicine, University of Magallanes, Casilla, Punta Arenas, Chile; Medical University of Vienna, AUSTRIA

## Abstract

In this study, we propose a novel three-criteria performance score to semiquantitatively classify the running style, the degree of involvement and compliance and the validity of electric shock count for rats exercising on a treadmill. Each score criterion has several style-marks that are based on the observational registry of male Sprague-Dawley rats running for 4–7 weeks. Each mark was given a score value that was averaged throughout a session-registry and resulting in a session score for each criterion, ranging from “0” score for a hypothetical “worst runner”, to score “1” for a hypothetical “perfect runner” rat. We found significant differences throughout a training program, thus providing evidence of sufficient sensitivity of this score to reflect the individual evolution of performance improvement in exercise capacity due to training. We hypothesize that this score could be correlated with other physiological or metabolic parameters, thus refining research results and further helping researchers to reduce the number of experimental subjects.

## Introduction

A significant amount of scientific literature on exercise sciences is based on experimental research involving laboratory rats, being motor-driven treadmill running, voluntary wheel running and swimming the most common types of exercise [1–6]. Although a marked difference has been reported on the exercise capacity of rats in relation to different strains [7–9], in most published work using treadmill training the mention of problems with the rats whilst carrying out the training sessions is absent [10–13]. This could lead to the assumption that all or much of the rats have been trained in the same extent by the end of a training session within each study. However, the natural proneness of rat for short voluntary running bursts [14] suggests that, if given the choice, it would run with recurrent breaks; unless, perhaps, if running away from a perceived danger. Thus, the expectancy of good habituation to continuous running on a treadmill, throughout a relatively long exercise session, might not be realistic from a biological point of view, especially at the beginning of a training period. Obviously, the rats can still be gradually trained to run, but the fact the rats finished a training session does not guarantee that all of them did the same training volume or equivalent intensity. Few published experiments reported on the different performance between individual rats when running. For instance, Arnold et al. (2014) described a protocol to select aged rats for an exercise protocol, arguing that not all animals are equally prone to run in a treadmill [15], while in a paper from Ferraresso et al. (2012) the authors specifically stated that they differentiated animals that ran voluntarily and animals that refused to ran in order to distribute the rodents among the different experimental groups [16]. However, this kind of information is usually omitted in experimental papers.

**Fig 1:**
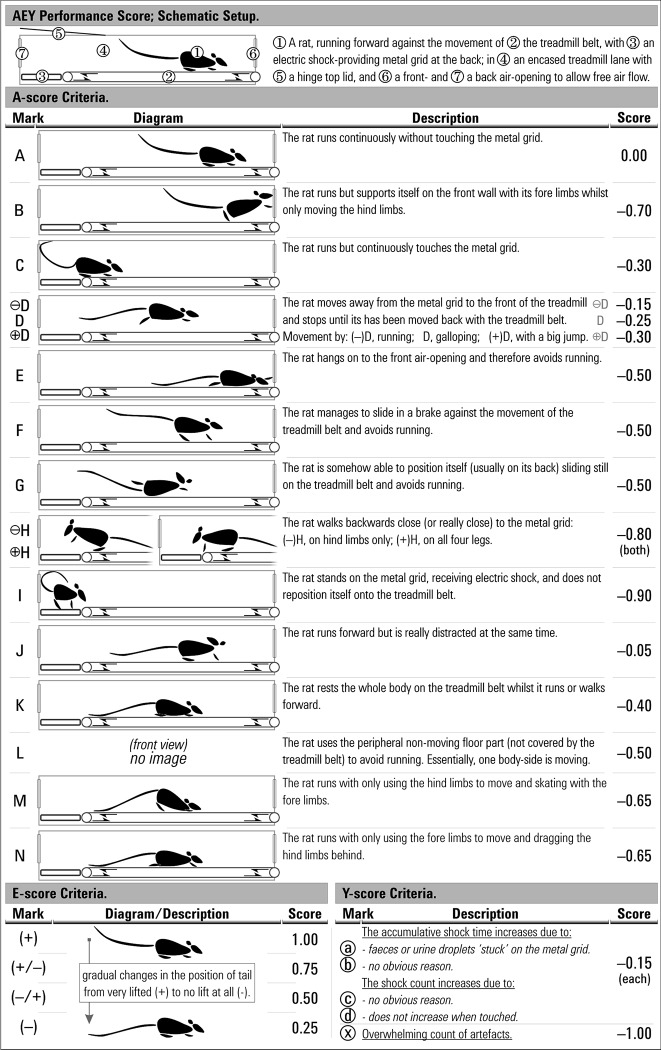
Schematic setup of the AEY performance score. The top section explains the setup of a treadmill diagram, followed by three sections for each score criteria: A, E and Y. The score criteria-sections have subsections (from left to right): (1) Mark, a registry note for the described style; (2) Diagram, a schematic drawing (if applicable); (3) Description, the characteristics are described; and (4) Score, the determined score value for further calculations.

Many treadmills for rats promote running by means of a light electric shock stimulus via the touch of a metal grid. A monitoring apparatus processes the input of the electric shock count with the related accumulating duration; useful to estimate how successfully a rat has been running. However, this setup has two potential problems: 1) the equipment cannot differentiate between a rat that is continuously touching the grid and e.g. a piece of faeces stuck on it; 2) when rats run on the treadmill, a marked majority tend to avoid running in many different ways. Fig 1 (marks B-N) describes evasive behaviours, which compromise compliance, to avoid running or electrical shock punishment [17]. Thus, the experimenter needs to be aware of these possible artefacts when using a treadmill.

Accepting the likelihood of all rats not running equally, or being unequally prone to run on a treadmill, leads to the question of how different qualitative running traits can be represented and how to quantify that. Assumption of equal performance and compliance for the same workload of all the animals in a training protocol is unrealistic. Indeed, if the training itself is important, also the training type is important; e.g. continuous vs. interval training or forced vs. spontaneous exercise. Subsequently, the fundamental questions should address the manner how the rats diverge in training and how this is reflected in the data, and address a systematic approach to determine those deviations.

Here we propose a system based on marks aimed to semiquantitatively assess the rat running style on a treadmill, and quantify this assessment with a score value, eventually leading to an average score. Thus, the main goal of this work is to develop and apply a semiquantitative scoring system to assess rat running compliance to an exercise protocol in a motorized treadmill. This score allows to “classify” the rats, which can serve as a critical factor for further physiological data interpretation. The score could also be used to compare the treadmill running performance of rats between different experimental conditions. Moreover, from an ethical point of view, and according to principles of the Three Rs (reduction, refinement and replacement), it is necessary to apply data tools that allow researchers to obtain the best from the experimental animals with which they are working. A vast majority of researchers simply discard animals that do not show ability to run on the treadmill, but the selected group might not be representative of the “normal” animal population. On the other hand, it certainly becomes an ethical issue if the selection of the experimental animals relies on discarding them, rather than refining the number of rats in the experiment from the onset.

## Material and methods

### Animals

All procedures were performed in accordance with the internal protocols of our laboratory, authorized by the University of Barcelona’s Ethical Committee for Animal Experimentation and ratified, in accordance with current Spanish legislation, by the Departament de d'Agricultura, Ramaderia, Pesca i Alimentació of Generalitat de Catalunya (file #8784). The score developed and presented in this article derived as a secondary observational registry from 130 male Sprague Dawley rats (strain: RjHan:SD, Janvier Labs, France). The animals were used in a primary experiment on the study of the recovery of induced skeletal muscle damage in the hind limbs of trained rats [18–21] in the framework of a research project approved by the Institutional Experimental Animal Ethics Committee. The experimental protocol required several weeks of previous conditional exercise training in a running treadmill and 1 day of exhaustive downhill exercise followed by different recovery interventions. All the rats used were males and started the training program at 6 weeks of age (body weight: 154 ± 8 g) in order to fulfil the requirements of the primary study, were housed at maximum 3 animals per cage (215×465×145 mm) and were fed standard diet (15-mm diameter granulates) and water ad libitum. The facility’s room temperature and relative humidity ranged between 20–25°C and 45–55% respectively. All the rats were regularly checked for stress signs judging from their physical appearance and body weight and received treatment fulfilling the National and European directives for the care of animal uses for scientific purposes [22].

### Instrumental

An encased five lane treadmill and its accompanying treadmill controller (LE 8710, Harvard Apparatus, United States) with an adjustable plane (5°/ramp-setting) from −15° to 25° was used to carry out the exercise training. An adjustable electric shock stimulator, ranging from 0.2–2.0 mA, discharged when animal contacted the metal grids behind the back end of the treadmill belt. The treadmill encasing, separating the lanes, with front and back wall air holes for each lane, enabled uncontrolled airflow inside it. For each lane, the monitor on the treadmill controller displayed the number of electric shocks generated, the accumulated time of electric shocks and the calculated distance considering the set velocity and the time lapsed, deducting the time spent receiving a shock. Speed adjustments affected collectively all the five lanes of the treadmill belt. Furthermore, the experimenters encouraged the rats to run with a light push or a sound to minimise the experience of excessive electric shocks, especially during the first days of habituation to exercise on the running treadmill.

### The three-criteria performance score

The score’s style code is based in three domains, each giving their own score value. These 3 criteria/scores are: 1) the running “attitude” (A), 2) a clue of the “endurance” (E), and 3) the gross “yield” (Y) provided by the electric shock count. Henceforward, the three-criteria performance score can be summarized as the AEY-score. More specifically, the criteria for the A-score concerns the physical positioning and actions of the rat whilst running (or trying to avoid running). The E-score concerns the position of the rat’s tail as an indicator of tiredness based on the effort to hold it up to avoid receiving an electric shock from the grid. The Y-score addresses the potential artefacts and problems considering the digital representation of the electric shock count (the sole insight usually considered of the rat performance in the running treadmill).

The development of the score was carried out in 2 stages: (a) the initial stage (55 rats) where different situations and characteristics were documented and the criteria for the scores were defined; and (b) the quantification stage (75 rats) where the defined criteria were systematically registered for each rat throughout every training session.

Fig 1 presents illustrative style diagrams and text explanations relative to the three domains of the score [A;E;Y] along with its corresponding marks and score values used for registering and quantifying during a training session. The experimental objective was to train the rats towards their best potential, recording the individual trends along the way. If it was needed and physically possible, the experimenters interfered with a rat’s unwanted styles by outlasting its determination. Relatively low treadmill speed (in the first days of habituation as part of the protocol) partially led to unfavourable running styles in some rats; hindering the development a continuous “proper” running (style A). Rats that demonstrated style B and E had no major issues with exercising and needed to be lightly pushed or pulled back onto four legs. Styles C, H and I represented rats that normally had high shock counts and needed an extra physical push to achieve a favourable positioning on the belt. Style D came across as a display of tiredness (or a sense of outwitting) by periodically stopping until almost touching (or fully touching) the shock grid and then quickly moving to the front of the treadmill. In styles F and G, the rat cleverly discovered that, by lightly touching its lane-sidewalls, enough resistance was provided to slide on the treadmill belt without moving towards the grid. Among styles E, F and G, the rat was equally inactive regarding the exercise; hence, all styles were later assigned the same quantification score, leaving the AEY-score open to take on board similar circumstances without major changes. Style J represented distracted or stressed rats (not necessarily “bad runners”) seeming susceptible to disrupting stimuli outside the treadmill. Styles K, M and N, normally occurred when rats were exercising to exhaustion; with style M prevailed on a downward slope. Style L is specific to treadmills where the belt does not cover the edge (e.g. within the two peripheral lanes of a multi-lane treadmill; a design flaw to be solved). In our case, a style L-rat supported the fore and hind limbs of the same body side onto the non-moving part, whilst the other limbs moved. The occurrence of style L was further minimised by rotating the lane positions between sessions during the first days of the training protocol.

### Quantifying the assessment

Each domain of the score had a maximal value of “1” that was considered for the “ideal runner” rat. The running styles or situations that were considered unfavourable would reduce that value towards the score floor of “0” for each part of the score. Thus, the more a trait compromised the animal’s compliance to the exercise protocol (to run continuously at 0.45 m/s for 30 min, see *Exercise Protocols* below), the more negative score was assigned to that trait.

For the A-score, the rat was given the roof value of “1” by default. Only one style was considered to represent the “ideal runner” (style A) and reduced this “1” by the value of “0” (i.e. no effect). Meanwhile, the other styles, having unfavourable traits of various degree, were given negative values (Fig 1, score column). Many of the unfavourable traits were visually different, although a similar negative value was assigned.

For the E-score (Fig 1), the value was applied directly with only four possible style assignations: between the most favourable, with the value of “1”, and the most unfavourable, with the value of “0.25”. The value of “0” was only applied if no registry was obtained during the registry period.

The Y-score was assigned the default value of “1” if no observation was registered. The 4 major types of observations (Fig 1), generally followed by a detailed description in the registry notes, were considered all equally influential and assigned the same value of “–0.15”. Each type was only counted once per registry period giving the lowest possible score of “0.4”. The Y-score therefore, served as an indicator for the underlying incidences. However, during registration, if some incidence was considered having a major effect on the monitor-readings, or continuously reoccurred, the score could be assigned with a “0” with the representative mark (Fig 1), to differentiate numerically from the lowest possible calculated score of “0.4”.

### Averaging out the scores

**Fig 2:**
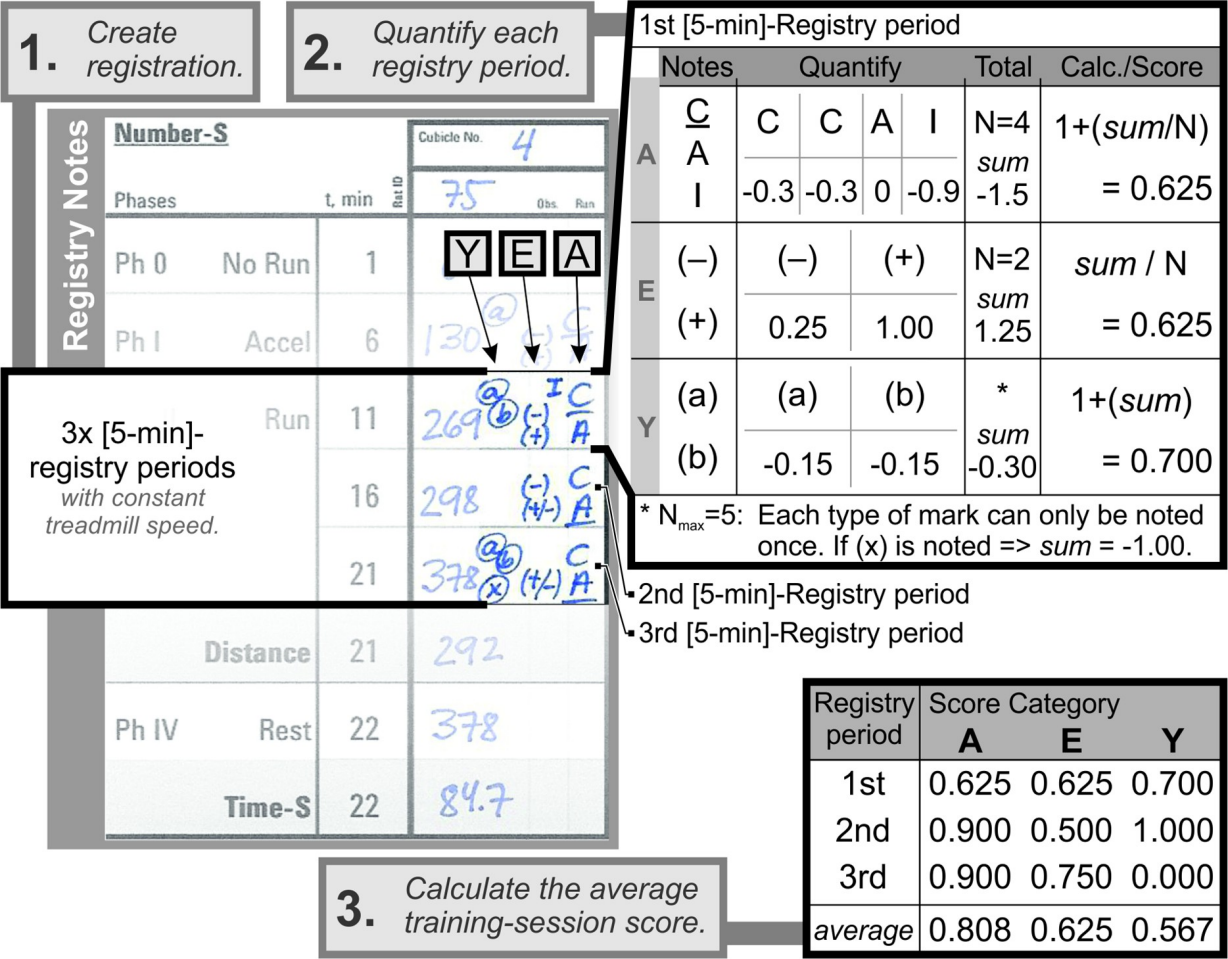
Representative registry notes. A representative registry note for one rat in a protocol with three 5-min registry periods at constant treadmill speed, where: (1) The observational notes during the training protocol are listed for each registry period; (2) the notes in each registry period are individually quantified for each part of the score; and (3) the average value for each part of the score are calculated between all the registry periods to obtain the average training-session AEY score of [0.808;0.625;0.567].

The running styles of each rat were registered with the corresponding marks for each of the particular score values (Fig 1) during all registry periods of a training session. As each registry period (5 min) could contain various styles registered, the notion of a dominant style (the underlined mark in the registry) was considered as having occurred at least twice (in A and E-domains). A representative registry form used in the semiquantitative assessment, with three different 5-min registry periods, is displayed in Fig 2 (Step 1).

For each domain of the score criteria (A, E and Y), within each individual registry period, the score values were calculated (Fig 2, Step 2) as:

$$A‐\mathrm{score}=1+\frac{\Sigma_{A‐\mathrm{mark}‐\mathrm{score}}}{n_{A‐\mathrm{mark}}}$$

$$E‐\mathrm{score}=\frac{\Sigma_{E‐\mathrm{mark}‐\mathrm{score}}}{n_{E‐\mathrm{mark}}}$$

$$Y‐\mathrm{score}=1+\Sigma_{Y‐\mathrm{mark}‐\mathrm{score}}$$

where, n_mark_ symbolises the number of marks notes; the Σ_mark-score_ represents the sum of mark-score values as defined in Fig 1, and the “1” represents a theoretical best value, when applied. Thereafter, the resulting scores were averaged out between all registry periods resulting in a training-session score for each rat (Fig 2, Step 3). The average training-session scores were further calculated to represent periods of several sessions and the standard deviations of these training-session scores were used to evaluate the variability between different rats of the same experimental group.

### Representation of the AEY score

The score should be represented as [A;E;Y], where a theoretical best rat would have [1;1:1] and the theoretical worst rat [0;0;0]. The score should be accompanied by detailed information about the training setup, including: (i) defined experimental period or duration; (ii) training day-frequency per week; (iii) training sessions per day, type of session and resting period between sessions (if applicable); and (iv) session time length (t), set speed (v), treadmill slope angle (θ). The average score can be calculated to represent different temporary stages, i.e.: a training day, a week of training, a general training program score, or a more extended-overall score such as a rat life-span score.

### Exercise protocols

All exercise sessions started with a 5-min warmup (not included in the session time length), to gradually reach a target speed. The rats trained for 29 days over the period of 7 weeks, training 5 days/week (except when noted below), never training on the weekends. They trained either 1 session/day (always around 09:00 h) or 2 session/day (latter session around 17:00 h), as continuous-training sessions.

Week 1 consisted of three consecutive training days before the weekend, 1 session/day (except 2 session/day the last day), with the gradual changes of: v = 0.30–0.34 m/s, t = 10–25 min/session (θ = 0°). Week 2 consisted of 2 session/day with the gradual changes of: v = 0.35–0.45 m/s, t = 30–32 min/session (θ = 0°).

Week 3 and 4 consisted of 2 session/day with v = 0.45 m/s, t = 30 min/session (θ = 0°). Day D (downhill protocol) consisted of 2 sessions with v = 0.55 m/s, t = until exhaustion (≈90 min) and 45 min/session (former and latter, respectively) and θ = −15°. This day was the 2nd day of week 5, were the 1st day was a resting day. Weeks 5, 6 and 7 consisted of 1 session/day (around 13:00 h) at v = 0.30 m/s, t = 15 min and θ = +5°, but only for one of the experimental groups during the muscle injury recovery period. Week 7 consisted of only two consecutive training days straight after the weekend.

### Statistical analysis

**Fig 3:**
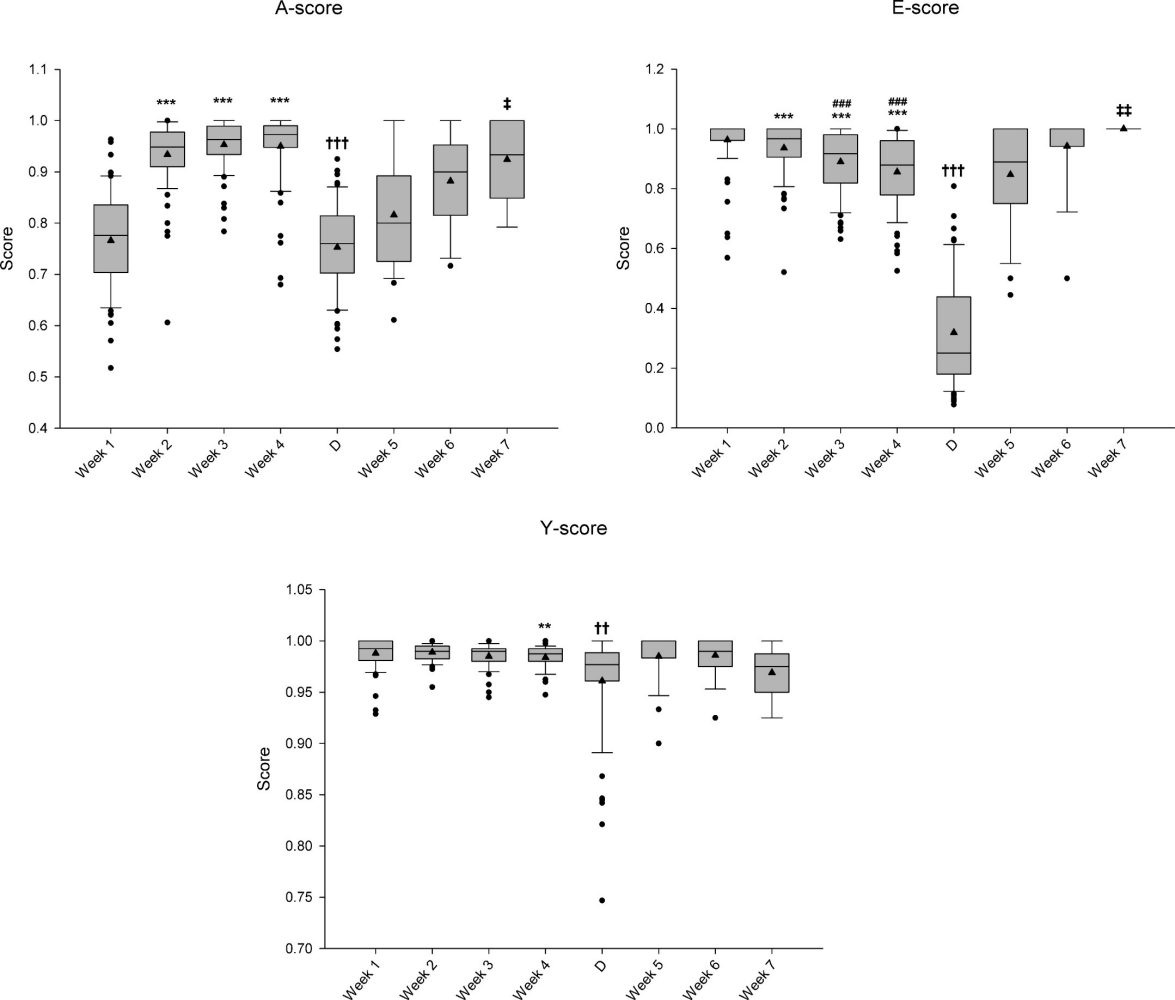
AEY Score along an exercise program. A box plot representation of the weekly average values for the 3 parts of the rat AEY performance score throughout a 7-week treadmill-training programme. On week 1 and 2 the rats (N = 75) carried out a gradual preconditioning training in preparation for a steady training on week 3 and 4. On week 5, 6 and 7 the rats (N = 27, 12 and 6, respectively) carried out light rehabilitation exercise after one day of downhill exhaustion exercise (N = 65) marked specifically as day D when commencing week 5. Statistically significant differences are indicated as follows: * vs. Week 1; # vs. Week 2; † vs. Week 4; ‡ vs. Week 5. One, two, and three repeated symbols correspond to P<0.05, P<0.01, and P< 0.001, respectively.

**Fig 4:**
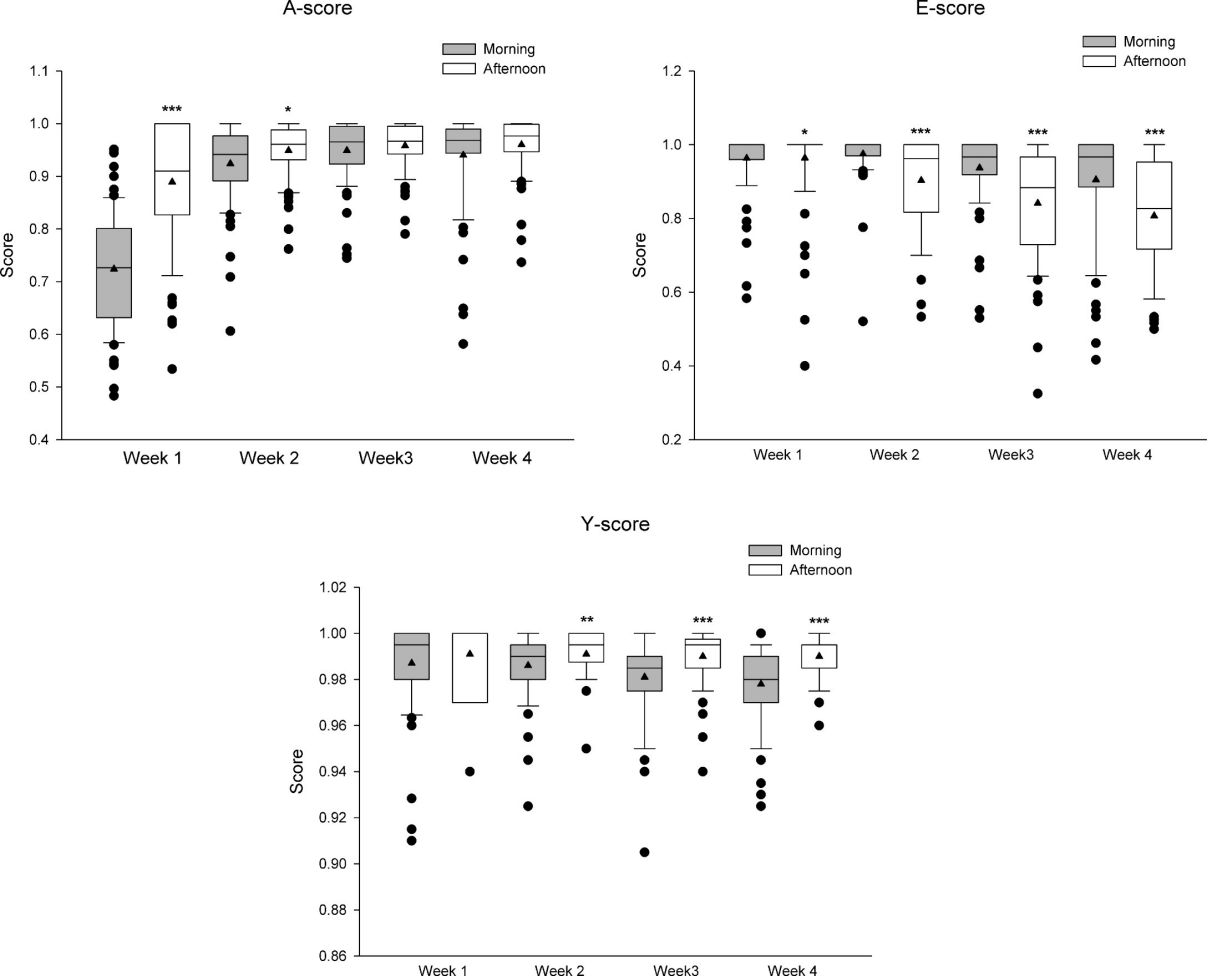
AEY Score comparison at two different daytimes. A box plot representation of the morning and afternoon session-average values for the 3 parts of the rat AEY performance score for 75 rats throughout the first four weeks of a 7-week treadmill-exercise programme. On week 1 and 2 the rats carried out a gradual preconditioning training in preparation for a steady training on week 3 and 4. Statistically significant differences are indicated as follows: * vs. Morning within the same week. One, two, and three repeated symbols correspond to P<0.05, P<0.01, and P< 0.001, respectively.

Data in Figs 3 and 4 are represented as box plots. The box represents the interquartile range and shows the first and the third quartiles, which are separated by the median. Black triangles represent the mean. Whisker end points represent the standard deviation, and the black circles represent outlier values. Scores between Week 1 –Week 4 and between Week 5 –Week 7 were statistically compared using a Kruskal-Wallis test followed by Dunn’s multiple comparison post hoc test. The Mann–Whitney U test was used to compare morning vs. afternoon scores within each week and to compare Week 4 vs. D. Statistical significance was considered when P<0.05.

## Results

Fig 3 presents the average score and standard deviation obtained throughout the entire training period, where each week, albeit with different count of training sessions, is separated with at least a weekend of rest. The A-score significantly increases after the first week showing a progressive lower dispersion trend. From day D until week 7, a similar pattern is observed, although starting from lower score values. Throughout weeks 1 to 4, the E-score follows an opposite trend to the A-score, reflected in significant decreasing mean values. Day D shows a marked and significant drop in mean values and the greatest deviation. Thereafter, the average level increases with a simultaneous deviation decrease. The Y-score appears as quite stable throughout the entire experimental period with a significant drop in the average at day D accompanied by increased deviation and, furthermore, a slight non-significant decrease on week 7 along with a greater deviation.

Fig 4 focuses on the first four weeks (training period), each separated by a weekend-rest, where the two daily training sessions (at morning and afternoon) are displayed separately. Whilst the overall weekly tendencies are similar in all three parts of the score, when comparing morning and afternoon mean values, the magnitude of the tendencies varies in A- and E- scores. The A-score of morning session is significantly lower than the score of afternoon session in the first two weeks. The opposite occurs with E-score, where the afternoon session-values are significantly lower over the 4 weeks. The Y-score shows a marked difference between the morning and the afternoon sessions.

## Discussion

### General considerations

The main goal of this paper was to develop a semiquantitative tool for an extensive and sensitive assessment of the exercise performance on a running treadmill- of laboratory rats. It is easy to design a scoring system only based on “good runners” rats, but running ability is highly variable. Surprisingly, in most published work, problems during habituation to run and performance heterogeneity among trained rats are not mentioned, maybe because most of the animals finally run relatively well or because researchers discard “bad runners”, which finally are used for sedentary or control groups. In any case, it is a fact that the contingency of being a “good runner rat” can be categorised. From our experience, some rats like running from the very beginning of the experiment and they cope well with the run as the speed is increased. Other rats appear putting much more attention on avoiding the shock grid than on forward running, and others continuously touch the grid and need a long time to become continuous runners. The question posed here is whether the score itself can semiquantitatively differentiate between these types of running rats and if AEY score correlates to the qualitative differences observed when they are monitored during the training sessions.

Whilst the criteria within the A-score have been quantified in accordance with an impact on the theoretical best style of running (mark A), the simple underlying mathematical design does not allow an average score to be directly back-traced to the dominant style/mark registered. That sort of evaluation requires looking at the actual registry and using different statistical approach. All the accumulative average-calculations render the back tracing-style estimation improbable. On the other hand, as the E-score focuses exclusively on the tail’s positioning as a sign of tiredness, its marks are more descriptive and correlate better with the actual score value they give. The assessment for the Y-score is different since it serves rather as a quality control index for the data output from the treadmill monitoring apparatus.

### Application of the AEY-score in different exercise protocols

We repeatedly noted that the rats caught up differently with the exercise on the running treadmill during the first training sessions. In Fig 3, this progression is reflected in the greatest deviation in the A-score on week 1, which progressively decreases until week 4. Simultaneously, the E-score demonstrates an increasing variation, suggesting a dissociation between the “skill” to achieve a continuous running style and the endurance of the animal. Even more, it can be hypothesized that as rats learn to run more continuously, they are more prone to fatigue probably because 1) the real ran distance increases; and 2) rat metabolism and anatomy is prepared for short voluntary running bursts instead of continuous speed [14]. The exercise sessions at day D are completely different; being a downhill running-to-exhaustion protocol, designed to induce muscle damage instead of a continuous running at constant slope and speed. During that protocol, a more A-score variety took place, which was reflected in a lower average A-score with greater underlying variation; and the E-score dropped significantly, as expected in a protocol designed to be extenuating. These results indicate that the AEY score is indeed able to discriminate between good and bad performance: during regular, moderate training (weeks 3 and 4) most animals perform well, obtaining scores close to 1. On the other hand, at day D the performance significantly decreased as a consequence of the exhaustive downhill running protocol. Indeed, a significant increase in the plasmatic concentration of muscle damage biomarkers creatine kinase-MM and myoglobin was found in a subgroup of animals sacrificed 24 h after day D [23], suggesting that the alterations in the AEY score had a physiological basis.

Conversely, during the subsequent weeks (active recovery period from week 5 to week 7), the exercise protocol was very light. However, despite the reduced speed and duration of these sessions, rats exhibited significantly lower A-score the first week after the downhill running-to-exhaustion protocol. This A-score decrease could be associated to muscle damage [23], which could hinder rat’s ability to exercise continuously due to compromised muscle function, soreness or pain. Thus, these results suggest that this tool is sensitive enough to discern between healthy and animals with eccentric exercise-induced muscle damage.

In general, it is likely that motor control memory plays a role in the success of a good A-score; frequent similar sessions might improve it whilst less frequent or different sessions do not. Perhaps the training time in relation to circadian and internal biorhythms is also a factor of considerable importance. Comparing the morning and afternoon sessions (Fig 4), there is a significant difference in the A-score in the first two weeks. Indeed, since rats are nocturnal animals, the lower A-score obtained in morning sessions it is not surprising. Nocturnal animals are more prone to perform physical activity (search of food, mating, exploration) during crepuscular and night hours, which could explain their better performance during the afternoon sessions. It could be hypothesized that the presence of more artefacts, faeces and urine drops contacting the metal grid (reflected by the lower Y-score) during the morning sessions could be due to a physiological stress response [24] as a consequence of the intrinsic circadian biorhythm disruption in these animals. Furthermore, some learning effect and habituation to the motorized treadmill could contribute to the better afternoon scores. Conversely, due to a relatively short resting period before the afternoon session, the E-score (representing tiredness) decreased.

Finally, as can be observed in Fig 3, the Y-score remained constant regardless of the degree of habituation to the treadmill (week 1 vs. week 4) and the intensity and duration of the exercise, which ranged from exhaustive (D) to light (weeks 5–7). Thus, parameters directly related only to electric shocks count should not be used in rat performance and compliance evaluation, but could serve as a good quality control indicator of training session.

### Limitations and advantages

Probably the most adverse aspect of the proposed score is the non-automated assessment, although it gets easier with practice. There are many calculations involved, albeit simple, and setting them up in a software programme such as Microsoft Excel is one way to work automatically through them. The main benefits, besides the pinpointing of potential outliers, are that the method provides a systematic and sensitive way to compare rats in different treatments or experimental conditions and even among different studies. Still, the assessment leading up to an individual AEY-score will always be subjected to some bias. In terms of future developments, the next step for the AEY score would be to correlate it with physiological representative parameters of exercise performance, such as VO_2_ measurements, in a similar way to the widely used Borg’s scale for rating perceived exertion in humans [25,26]. This could either reinforce our style-score correlation or shed a new light on it. Perhaps it is difficult and unrealistic to get a %VO_2_max correlation with specific running styles, but it could correlate with our 0–1 scale in either or both the A- and E-score. Moreover, this and other physiological measurements would be needed to further assess the validity of the proposed tool. Additionally, this score system is open enough to be applied to different strains and age. However, because we do not have tested this tool in these cases, one should empirically check if the AEY-score needs any adjustments, especially regarding the running style. For instance, it is plausible that healthy but aged rats would be unable to keep running with an A-style for a given speed and duration, or that an specific running behaviour would be more prevalent in certain conditions. In these cases, experimenters could decide to modify the scores and classifications to better fit their experimental conditions.

## Conclusion

As an inexpensive tool relying on the experimenter’s observational capacity, we have suggested the three-part AEY score is a good method to assess and describe the laboratory rat performance capacity during exercise on a running treadmill in a semiquantifiable manner. This would be useful for considering and correlating the sample dispersion with other physiological or anatomical parameters and, moreover, to obtain the best from the animals in question, thus contributing to promoting the principles of the 3Rs (Replacement, Reduction and Refinement) in the use of experimental animals.

## Supporting information

S1 File RawDataAEYScore. This database contains the registered data and calculations for the exercise style of each rat on a running treadmill according to the descriptions in Fig 1 and as reflected in the sheet 1 labelled as “Styles”. The procedure was applied in four distinct phases of a higher scale project designed for the study the effects of different interventions for skeletal muscle recovery after injury induced by forced eccentric exercise in trained rats. This phases were: a two weeks habituation to treadmill exercise period (sheet 2 “Habit”), a four weeks of exercise training period (sheet 3 “Training”), a day of two sessions (4 hours of break) of downhill exercise until exhaustion (sheet 4 “DH Day”), and a rehabilitation period of 21 days (sheet 5”Rehab”). Pooled total data are available in a whole dataset (sheet 6 “Pool”).
(XLSX)
